# Supplementary material for: From complexity to clarity: How AI enhances perceptions of scientists and the public's understanding of science
Source: PNAS Nexus. 2024 Sep 6;3(9):pgae387. doi: 10.1093/pnasnexus/pgae387 (PMC11406778; doi:10.1093/pnasnexus/pgae387)
Supplement: pgae387_Supplementary_Data [file pgae387_supplementary_data.docx]

**
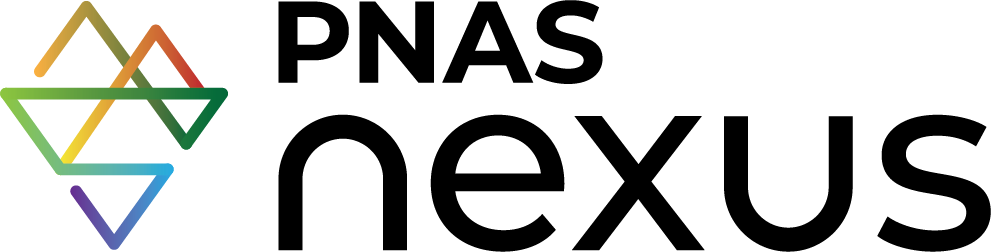
**

**Supplementary Information for**

From Complexity to Clarity: How AI Enhances Perceptions of Scientists and the Public’s Understanding of Science

David M. Markowitz

David M. Markowitz

Email: dmm@msu.edu

**This PDF file includes:**

Table of contents

Supplementary results

Stimuli

References

Table of Contents

[Table S1: Descriptive Statistics and Correlation Matrix for Study 1a 3](#_Toc175924569)

[Table S2: Exploratory LIWC Results Across GPT and PNAS Significance Statements: Study 1b 4](#_Toc175924570)

[Table S3: Exploratory Results With Additional LIWC Covariates: Study 1b 8](#_Toc175924571)

[Table S4: Themes Extracted in Study 1b Using the Meaning Extraction Method 9](#_Toc175924572)

[Additional Findings Using Thematic Results 10](#_Toc175924573)

[Table S5: Multivariate Results Controlling For All Content Dimensions: Study 1b 11](#_Toc175924574)

[Stimuli in Study 2 13](#_Toc175924575)

[Table S6: Descriptive Statistics and Correlation Matrix for Study 2 16](#_Toc175924576)

[Verbatim Prompts for Study 3 17](#_Toc175924577)

[Table S7: Descriptive Statistics and Correlation Matrix for Study 3 18](#_Toc175924578)

[References 19](#_Toc175924579)

# **Table S1: Descriptive Statistics and Correlation Matrix for Study 1a**

| Variable | *M* | *SD* | 1 | 2 |
| --- | --- | --- | --- | --- |
|  |  |  |  |  |
| 1. Common words | 68.78 | 6.94 |  |  |
|  |  |  |  |  |
| 2. Analytic writing | 93.33 | 6.79 | -.18** |  |
|  |  |  | [-.19, -.18] |  |
|  |  |  |  |  |
| 3. Readability | 12.73 | 13.22 | .24** | -.07** |
|  |  |  | [.24, .25] | [-.08, -.06] |
|  |  |  |  |  |

*Note*. ** *p* < .01. Numbers in brackets are 95% Confidence Intervals

# **Table S2: Exploratory LIWC Results Across GPT and PNAS Significance Statements: Study 1b**

|  | mean_GPT | mean_PNAS | *df* | *t* | *p* |
| --- | --- | --- | --- | --- | --- |
| WC | 98.99 | 110.47 | 1305.95 | -18.88 | .000 |
| Analytic | 92.73 | 92.32 | 1587.65 | 1.16 | .246 |
| Clout | 38.16 | 49.10 | 1533.24 | -13.40 | .000 |
| Authentic | 34.82 | 33.08 | 1597.35 | 1.36 | .174 |
| Tone | 42.47 | 33.12 | 1587.53 | 8.22 | .000 |
| WPS | 22.52 | 23.43 | 1273.03 | -4.36 | .000 |
| BigWords | 41.59 | 41.56 | 1561.11 | 0.10 | .922 |
| Dic | 75.53 | 69.84 | 1478.73 | 17.31 | .000 |
| Linguistic | 51.34 | 48.44 | 1424.84 | 10.97 | .000 |
| function. | 41.21 | 39.04 | 1468.51 | 10.39 | .000 |
| pronoun | 7.05 | 5.43 | 1571.95 | 15.83 | .000 |
| ppron | 0.79 | 1.97 | 1499.17 | -21.50 | .000 |
| i | 0.02 | 0.04 | 1454.97 | -2.04 | .042 |
| we | 0.44 | 1.53 | 1339.99 | -26.49 | .000 |
| you | 0.00 | 0.00 | 799.00 | -1.00 | .318 |
| shehe | 0.00 | 0.00 | 1588.10 | 0.06 | .955 |
| they | 0.32 | 0.37 | 1595.57 | -1.61 | .107 |
| ipron | 6.26 | 3.45 | 1594.07 | 33.11 | .000 |
| det | 16.14 | 12.33 | 1553.59 | 25.00 | .000 |
| article | 9.07 | 7.25 | 1592.86 | 14.10 | .000 |
| number | 1.21 | 2.37 | 1295.96 | -9.43 | .000 |
| prep | 14.22 | 14.45 | 1569.86 | -1.89 | .059 |
| auxverb | 3.65 | 3.95 | 1581.12 | -3.30 | .001 |
| adverb | 2.80 | 2.51 | 1597.05 | 3.56 | .000 |
| conj | 4.47 | 4.88 | 1577.54 | -4.14 | .000 |
| negate | 0.27 | 0.25 | 1597.95 | 0.74 | .460 |
| verb | 6.12 | 6.43 | 1572.38 | -2.52 | .012 |
| adj | 7.38 | 6.18 | 1578.81 | 8.35 | .000 |
| quantity | 2.05 | 3.10 | 1510.77 | -9.84 | .000 |
| Drives | 4.01 | 4.68 | 1571.93 | -5.34 | .000 |
| affiliation | 0.79 | 1.88 | 1547.97 | -17.98 | .000 |
| achieve | 1.82 | 1.24 | 1582.21 | 7.45 | .000 |
| power | 1.77 | 1.72 | 1592.95 | 0.62 | .534 |
| Cognition | 15.71 | 11.77 | 1587.35 | 19.50 | .000 |
| allnone | 0.05 | 0.14 | 1255.12 | -5.87 | .000 |
| cogproc | 15.66 | 11.61 | 1588.18 | 20.10 | .000 |
| insight | 5.02 | 3.37 | 1572.49 | 14.07 | .000 |
| cause | 3.58 | 3.43 | 1597.59 | 1.45 | .147 |
| discrep | 1.48 | 0.71 | 1565.26 | 15.92 | .000 |
| tentat | 1.41 | 1.24 | 1581.69 | 2.75 | .006 |
| certitude | 0.05 | 0.14 | 1326.10 | -5.77 | .000 |
| differ | 2.61 | 2.75 | 1586.29 | -1.54 | .124 |
| memory | 0.06 | 0.06 | 1597.33 | 0.20 | .845 |
| Affect | 2.70 | 2.22 | 1598.00 | 4.87 | .000 |
| tone_pos | 2.03 | 1.43 | 1592.28 | 8.15 | .000 |
| tone_neg | 0.63 | 0.74 | 1588.98 | -1.97 | .049 |
| emotion | 0.26 | 0.33 | 1596.23 | -1.45 | .148 |
| emo_pos | 0.03 | 0.06 | 1257.10 | -2.06 | .039 |
| emo_neg | 0.20 | 0.22 | 1597.90 | -0.62 | .537 |
| emo_anx | 0.10 | 0.10 | 1522.52 | 0.29 | .768 |
| emo_anger | 0.03 | 0.04 | 1563.81 | -1.34 | .182 |
| emo_sad | 0.03 | 0.03 | 1595.01 | -0.16 | .874 |
| swear | 0.00 | 0.01 | 874.67 | -1.27 | .206 |
| Social | 4.94 | 5.46 | 1589.18 | -3.39 | .001 |
| socbehav | 2.81 | 2.26 | 1596.59 | 6.07 | .000 |
| prosocial | 0.38 | 0.45 | 1563.28 | -1.70 | .089 |
| polite | 0.02 | 0.05 | 1435.79 | -1.79 | .073 |
| conflict | 0.07 | 0.11 | 1441.75 | -2.68 | .007 |
| moral | 0.03 | 0.04 | 1578.15 | -1.49 | .137 |
| comm | 0.93 | 0.67 | 1593.56 | 5.22 | .000 |
| socrefs | 1.98 | 3.05 | 1574.41 | -10.86 | .000 |
| family | 0.07 | 0.08 | 1570.70 | -0.61 | .543 |
| friend | 0.01 | 0.01 | 1594.77 | 0.14 | .891 |
| female | 0.04 | 0.06 | 1485.52 | -1.12 | .264 |
| male | 0.08 | 0.12 | 1526.72 | -1.72 | .085 |
| Culture | 0.79 | 0.94 | 1580.43 | -2.37 | .018 |
| politic | 0.16 | 0.19 | 1596.28 | -1.10 | .272 |
| ethnicity | 0.02 | 0.03 | 1506.33 | -0.76 | .447 |
| tech | 0.60 | 0.71 | 1573.45 | -2.01 | .044 |
| Lifestyle | 4.30 | 2.52 | 1592.41 | 17.62 | .000 |
| leisure | 0.22 | 0.12 | 1516.54 | 4.15 | .000 |
| home | 0.03 | 0.06 | 1333.17 | -2.38 | .018 |
| work | 3.78 | 1.97 | 1593.32 | 19.64 | .000 |
| money | 0.14 | 0.20 | 1527.35 | -2.03 | .042 |
| relig | 0.17 | 0.20 | 1581.28 | -1.16 | .246 |
| Physical | 4.25 | 3.58 | 1593.06 | 3.26 | .001 |
| health | 2.99 | 2.34 | 1592.40 | 3.85 | .000 |
| illness | 1.53 | 1.25 | 1585.49 | 2.51 | .012 |
| wellness | 0.14 | 0.12 | 1597.82 | 0.51 | .610 |
| mental | 0.04 | 0.03 | 1511.15 | 0.55 | .580 |
| substances | 0.02 | 0.03 | 1564.37 | -0.50 | .618 |
| sexual | 0.07 | 0.07 | 1591.82 | 0.10 | .917 |
| food | 0.19 | 0.19 | 1583.12 | -0.13 | .897 |
| death | 0.08 | 0.10 | 1581.02 | -0.79 | .432 |
| need | 0.56 | 0.43 | 1594.47 | 3.59 | .000 |
| want | 0.00 | 0.02 | 1162.34 | -2.42 | .016 |
| acquire | 0.12 | 0.21 | 1506.21 | -3.62 | .000 |
| lack | 0.08 | 0.14 | 1542.63 | -3.00 | .003 |
| fulfill | 0.04 | 0.11 | 1365.72 | -5.17 | .000 |
| fatigue | 0.00 | 0.00 | 1229.55 | -0.40 | .686 |
| reward | 0.16 | 0.21 | 1592.91 | -1.89 | .058 |
| risk | 0.39 | 0.31 | 1544.62 | 1.92 | .056 |
| curiosity | 1.90 | 0.33 | 1512.69 | 39.81 | .000 |
| allure | 1.28 | 1.82 | 1551.79 | -7.98 | .000 |
| Perception | 7.88 | 8.64 | 1583.42 | -4.44 | .000 |
| attention | 0.26 | 0.34 | 1579.36 | -2.68 | .007 |
| motion | 0.75 | 0.88 | 1564.76 | -2.33 | .020 |
| space | 5.81 | 6.24 | 1581.70 | -3.31 | .001 |
| visual | 0.88 | 0.90 | 1597.21 | -0.41 | .680 |
| auditory | 0.05 | 0.08 | 1484.64 | -1.10 | .272 |
| feeling | 0.17 | 0.23 | 1598.00 | -1.48 | .140 |
| time | 2.64 | 2.51 | 1594.31 | 1.25 | .213 |
| focuspast | 1.20 | 1.49 | 1589.19 | -4.46 | .000 |
| focuspresent | 2.70 | 3.07 | 1565.82 | -4.84 | .000 |
| focusfuture | 1.14 | 0.71 | 1597.21 | 8.75 | .000 |
| Conversation | 0.03 | 0.10 | 1128.92 | -3.24 | .001 |
| netspeak | 0.01 | 0.06 | 840.53 | -3.67 | .000 |
| assent | 0.00 | 0.02 | 868.71 | -1.94 | .053 |
| nonflu | 0.02 | 0.04 | 1421.95 | -0.91 | .363 |
| filler | 0.00 | 0.00 | 799.00 | -1.00 | .318 |
| AllPunc | 12.50 | 13.87 | 1437.58 | -7.04 | .000 |
| Period | 4.52 | 4.54 | 1299.85 | -0.42 | .674 |
| Comma | 4.96 | 4.32 | 1567.31 | 6.32 | .000 |
| QMark | 0.00 | 0.03 | 799.00 | -4.40 | .000 |
| Exclam | 0.00 | 0.00 | NA | NA | NA |
| Apostro | 0.38 | 0.13 | 1205.21 | 8.10 | .000 |
| OtherP | 2.63 | 4.85 | 1324.68 | -13.85 | .000 |
| Emoji | 0.00 | 0.00 | NA | NA | NA |

# **Table S3: Exploratory Results With Additional LIWC Covariates: Study 1b**

| DV: Simplicity Index | | | | |
| --- | --- | --- | --- | --- |
| Variable | *B* | *SE* | *t* | *p* |
| (Intercept) | -2.76 | 0.20 | -13.75 | < .001 |
| Text type: PNAS | -0.35 | 0.10 | -3.57 | < .001 |
| Political speech | 0.13 | 0.07 | 2.03 | .043 |
| Affect | 0.18 | 0.02 | 8.02 | < .001 |
| Cognition | 0.16 | 0.01 | 14.27 | < .001 |
| Physical references | 0.09 | 0.01 | 7.88 | < .001 |
|  |  |  |  |  |
| DV: Common Words | | | | |
| Variable | *B* | *SE* | *t* | *p* |
| (Intercept) | 60.67 | 0.63 | 96.60 | < .001 |
| Text type: PNAS | -2.43 | 0.31 | -7.92 | < .001 |
| Political speech | 1.32 | 0.21 | 6.33 | < .001 |
| Affect | 0.79 | 0.07 | 11.15 | < .001 |
| Cognition | 0.66 | 0.03 | 19.29 | < .001 |
| Physical references | 0.52 | 0.03 | 15.41 | < .001 |
|  |  |  |  |  |
| DV: Analytic Writing | | | | |
| Variable | *B* | *SE* | *t* | *p* |
| (Intercept) | 102.25 | 0.79 | 129.32 | < .001 |
| Text type: PNAS | -2.68 | 0.39 | -6.94 | < .001 |
| Political speech | -0.33 | 0.26 | -1.28 | .202 |
| Affect | -0.36 | 0.09 | -4.07 | < .001 |
| Cognition | -0.52 | 0.04 | -12.24 | < .001 |
| Physical references | -0.06 | 0.04 | -1.40 | .162 |
|  |  |  |  |  |
| DV: Readability | | | | |
| Variable | *B* | *SE* | *t* | *p* |
| (Intercept) | 18.74 | 1.47 | 12.71 | < .001 |
| Text type: PNAS | -5.00 | 0.72 | -6.94 | < .001 |
| Political speech | -1.25 | 0.49 | -2.55 | .011 |
| Affect | 0.27 | 0.17 | 1.63 | .104 |
| Cognition | -0.12 | 0.08 | -1.52 | .129 |
| Physical references | 0.05 | 0.08 | 0.68 | .499 |

# **Table S4: Themes Extracted in Study 1b Using the Meaning Extraction Method**

| Component 1: | | Component 2: | | Component 3: | | Component 4: | | Component 5: | | | Component 6: | | | Component 7: | | | Component 8: | | |  |
| --- | --- | --- | --- | --- | --- | --- | --- | --- | --- | --- | --- | --- | --- | --- | --- | --- | --- | --- | --- | --- |
| Significance | | Insights | | Cruciality | | Implications | | Results | | | Gene expression | | | Methods | | | Cancer | | |  |
| λ | % | λ | % | λ | % | λ | % | | λ | % | | λ | % | | λ | % | | λ | % | |
| 4.49 | 3.30 | 2.36 | 1.74 | 2.24 | 1.65 | 2.23 | 1.64 | | 2.20 | 1.62 | | 2.07 | 1.52 | | 1.91 | 1.40 | | 1.85 | 1.636 | |
| Word | Loading | Word | Loading | Word | Loading | Word | Loading | | Word | Loading | | Word | Loading | | Word | Loading | | Word | Loading | |
| research significant insights | 0.950 | reveals | 0.805 | crucial role | 0.820 | implications | 0.772 | | findings suggest | 0.901 | | expression | 0.764 | | presents | 0.716 | | patients | 0.598 | |
| significant insights | 0.949 | study | 0.350 | crucial | 0.707 | implications understanding | 0.730 | | suggest | 0.882 | | gene | 0.733 | | method | 0.705 | | effective | 0.562 | |
| research significant | 0.915 | insights | 0.265 | role | 0.577 | important | 0.488 | | findings | 0.576 | | genes | 0.643 | | approach | 0.511 | | treatments | 0.486 | |
| insights | 0.655 |  |  | process | 0.238 | understanding | 0.439 | |  |  | |  |  | |  |  | | cancer | 0.463 | |
| significant | 0.638 |  |  |  |  |  |  | |  |  | |  |  | |  |  | | treatment | 0.425 | |
| research | 0.363 |  |  |  |  |  |  | |  |  | |  |  | |  |  | |  |  | |
| study reveals | 0.238 |  |  |  |  |  |  | |  |  | |  |  | |  |  | |  |  | |

*Note*. Components are dominant themes extracted using the Meaning Extraction Method. Based on prior work (1–3), the number of themes to extract were based on variance explained, thematic interpretability, and scree plot evidence. Components were saved as regression weights for analyses reported in this supplement. Unigrams (single words), bigrams (two-word phrases), and trigrams (three-word phrases) were extracted using this process. For words to be retained in this analysis, they must have appeared in at least 5% of the texts and each text received a score of 1 (presence) or 0 (absence) to indicate if a word was represented.

# **Additional Findings Using Thematic Results**

It is an open question if in certain fields, AI-generated summaries may be worse than human-written summaries. To evaluate this question, component scores were used from the thematic extraction analyses and they were associated with a composite variable of simplicity based on the linguistic dimensions of interest (i.e., common words, analytic writing, readability). Specifically, all variables were standardized (z-scored) and then the following formula was applied: Common words + readability - Analytic writing. High scores on this composite are linguistically simpler than low scores, generally reflected AI text as reported in Study 1b.

Bivariate correlations between the simplicity composite and each component revealed that the more that papers focused on gene expression (Component 6 in the supplement), the less that the writing style reflected AI/simple writing (*r* = -.065, *p* = .009). For all other components, the relationship between simplicity/AI writing and component scores were positive or not statistically significant.

# **Table S5: Multivariate Results Controlling For All Content Dimensions: Study 1b**

| DV: Simplicity Index | | | | |
| --- | --- | --- | --- | --- |
| Variable | *B* | *SE* | *t* | *p* |
| (Intercept) | -2.60 | 0.21 | -12.60 | < .001 |
| Text type: PNAS | -0.56 | 0.12 | -4.56 | < .001 |
| Political speech | 0.13 | 0.07 | 1.97 | .050 |
| Affect | 0.17 | 0.02 | 7.45 | < .001 |
| Cognition | 0.16 | 0.01 | 14.18 | < .001 |
| Physical references | 0.07 | 0.01 | 6.18 | < .001 |
| Component 1 | -0.12 | 0.05 | -2.54 | .011 |
| Component 2 | -0.07 | 0.05 | -1.46 | .144 |
| Component 3 | 0.00 | 0.04 | 0.07 | .943 |
| Component 4 | -0.05 | 0.05 | -1.17 | .242 |
| Component 5 | 0.06 | 0.04 | 1.26 | .207 |
| Component 6 | -0.04 | 0.04 | -0.85 | .399 |
| Component 7 | -0.17 | 0.05 | -3.86 | < .001 |
| Component 8 | 0.11 | 0.05 | 2.29 | .022 |

| DV: Common Words | | | | |
| --- | --- | --- | --- | --- |
| Variable | *B* | *SE* | *t* | *p* |
| (Intercept) | 60.86 | 0.65 | 93.84 | < .001 |
| Text type: PNAS | -2.72 | 0.39 | -7.04 | < .001 |
| Political speech | 1.32 | 0.21 | 6.31 | < .001 |
| Affect | 0.78 | 0.07 | 10.99 | < .001 |
| Cognition | 0.66 | 0.03 | 18.97 | < .001 |
| Physical references | 0.50 | 0.04 | 13.56 | < .001 |
| Component 1 | 0.16 | 0.15 | 1.03 | .305 |
| Component 2 | -0.26 | 0.15 | -1.76 | .079 |
| Component 3 | 0.08 | 0.14 | 0.57 | .570 |
| Component 4 | -0.16 | 0.14 | -1.10 | .271 |
| Component 5 | 0.02 | 0.14 | 0.14 | .891 |
| Component 6 | -0.15 | 0.14 | -1.10 | .274 |
| Component 7 | -0.47 | 0.14 | -3.33 | .001 |
| Component 8 | 0.13 | 0.15 | 0.83 | .405 |

| DV: Analytic Writing | | | | |
| --- | --- | --- | --- | --- |
| Variable | *B* | *SE* | *t* | *p* |
| (Intercept) | 101.22 | 0.81 | 124.55 | < 2e-16 |
| Text type: PNAS | -1.51 | 0.48 | -3.12 | .002 |
| Political speech | -0.33 | 0.26 | -1.28 | .201 |
| Affect | -0.33 | 0.09 | -3.69 | .000 |
| Cognition | -0.50 | 0.04 | -11.45 | < 2e-16 |
| Physical references | -0.05 | 0.05 | -1.17 | .242 |
| Component 1 | 0.80 | 0.19 | 4.22 | .000 |
| Component 2 | 0.26 | 0.19 | 1.38 | .168 |
| Component 3 | 0.49 | 0.18 | 2.76 | .006 |
| Component 4 | -0.26 | 0.18 | -1.45 | .148 |
| Component 5 | 0.06 | 0.17 | 0.33 | .740 |
| Component 6 | 0.10 | 0.17 | 0.56 | .576 |
| Component 7 | 0.49 | 0.18 | 2.76 | .006 |
| Component 8 | -0.07 | 0.19 | -0.38 | .702 |

| DV: Readability | | | | |
| --- | --- | --- | --- | --- |
| Variable | *B* | *SE* | *t* | *p* |
| (Intercept) | 18.58 | 1.52 | 12.26 | < .001 |
| Text type: PNAS | -5.09 | 0.90 | -5.64 | < .001 |
| Political speech | -1.30 | 0.49 | -2.66 | .008 |
| Affect | 0.18 | 0.17 | 1.10 | .272 |
| Cognition | -0.06 | 0.08 | -0.71 | .477 |
| Physical references | -0.06 | 0.09 | -0.71 | .479 |
| Component 1 | -0.43 | 0.35 | -1.20 | .231 |
| Component 2 | 0.04 | 0.35 | 0.11 | .911 |
| Component 3 | 0.78 | 0.33 | 2.36 | .018 |
| Component 4 | -0.89 | 0.34 | -2.62 | .009 |
| Component 5 | 0.79 | 0.32 | 2.45 | .014 |
| Component 6 | -0.03 | 0.32 | -0.10 | .918 |
| Component 7 | -0.53 | 0.33 | -1.59 | .113 |
| Component 8 | 1.07 | 0.35 | 3.04 | .002 |

*Note*. All component numbers correspond to the components represented in the section titled “Themes Extracted in Study 1b Using the Meaning Extraction Method.”

# **Stimuli in Study 2**

Pair 1: GPT

*This research provides new insights into the complex interactions between adenosine A2A receptors (A2AR) and dopamine D2 receptors (D2R), which play a crucial role in regulating brain function. The study reveals previously unknown mechanisms within these receptor interactions that can decrease the effectiveness of D2R. Interestingly, these effects disappear when both agonists and antagonists are present. This research also suggests that high concentrations of A2AR antagonists can act as agonists, reducing D2R function in the brain. These findings could have significant implications for understanding and potentially treating neurological disorders related to these receptors.*

Pair 1: PNAS

*G protein-coupled receptors (GPCRs) constitute the largest plasma membrane protein family involved in cell signaling. GPCR homodimers are predominant species, and GPCR heteromers likely are constituted by heteromers of homodimers. The adenosine A2A receptor (A2AR)-dopamine D2 receptor (D2R) heteromer is a target for the nonselective adenosine receptor antagonist caffeine. This study uncovers allosteric modulations of A2AR antagonists that mimic those of A2AR agonists, challenging the traditional view of antagonists as inactive ligands. These allosteric modulations disappear when agonist and antagonist are coadministered, however. A model is proposed that considers A2AR-D2R heteromers as heterotetramers, constituted by A2AR and D2R homodimers. The model predicted that high concentrations of A2AR antagonists would behave as A2AR agonists and decrease D2R function in the brain.*

Pair 2: GPT

*This research challenges existing thermal-mechanical models of subduction zones, which are areas where one tectonic plate moves under another. The study suggests that these models underestimate the temperatures at certain depths, which has implications for our understanding of geological phenomena such as metamorphic reactions and fault-slip events. The researchers propose that an additional heat source, likely shear heating, is needed to explain the higher temperatures. This finding could change our understanding of the conditions under which rocks are formed and moved in these zones. It also suggests that the rocks we see on the surface may not be representative of the conditions in younger, hotter subduction zones.*

Pair 2: PNAS

*Thermal structure controls numerous aspects of subduction zone metamorphism, rheology, and melting. Many thermal models assume small or negligible coefficients of friction and underpredict pressure–temperature (P–T) conditions recorded by subduction zone metamorphic rocks by hundreds of degrees Celsius. Adding shear heating to thermal models simultaneously reproduces surface heat flow and the P–T conditions of exhumed metamorphic rocks. Hot dry rocks are denser than cold wet rocks, so rocks from young-hot subduction systems are denser and harder to exhume through buoyancy. Thus, the metamorphic record may underrepresent hot-young subduction and overrepresent old-cold subduction.*

Pair 3: GPT

*This research provides significant insights into the behavior of heparan sulfates during sepsis, a life-threatening condition caused by the body's response to an infection. The study reveals that these molecules are rapidly cleared from the bloodstream and selectively penetrate the hippocampus, a region of the brain involved in memory and learning. This suggests that heparan sulfates could have functional consequences in this brain region during sepsis. Understanding these processes could potentially lead to new therapeutic strategies for managing sepsis and mitigating its effects on the brain.*

Pair 3: PNAS

*Sepsis results in the heparanase-mediated release of heparan sulfate oligosaccharides from the endothelial glycocalyx. In human sepsis patients, these released heparan sulfate oligosaccharides have been shown to be rich in highly sulfated domains, and their presence was associated with moderate or severe cognitive impairment. The current study uses a murine sepsis model to show that an exogenously administered highly sulfated 13C-labeled heparan sulfate oligosaccharide, rich in highly sulfated domains, selectively targets the hippocampus. This selective targeting suggests that heparan sulfate sequestering of brain-derived neurotrophic factor in the hippocampus may impact spatial memory formation. A therapeutic strategy for selectively protecting cognition in septic patients might be developed through targeting these heparan sulfate oligosaccharides, rich in highly sulfated domains.*

Pair 4: GPT

*This research provides significant insights into the relationship between oxidative stress and heart disease. It demonstrates that oxidative stress can modify the protein titin, which is crucial for heart muscle elasticity. Specifically, the study shows that oxidative stress can cause changes in the distal titin spring region, affecting the passive force in human heart cells and potentially leading to heart disease. This research suggests a new mechanism for how oxidative stress can contribute to heart disease, offering potential new targets for therapeutic intervention. Understanding this process could lead to new treatments for heart disease, a leading cause of death worldwide.*

Pair 4: PNAS

*Titin oxidation alters titin stiffness, which greatly contributes to overall myocardial stiffness. This stiffness is frequently increased in heart disease, such as diastolic heart failure. We have quantified the degree of oxidative titin changes in several murine heart and skeletal muscle models exposed to oxidant stress and mechanical load. Importantly, strain enhances in vivo oxidation of titin in the elastic region, but not the inextensible segment. The functional consequences include oxidation type-dependent effects on cardiomyocyte stiffness, titin-domain folding, phosphorylation, and inter-titin interactions. Thus, oxidative modifications stabilize the titin spring in a dynamic and reversible manner and help propagate changes in titin-based myocardial stiffness. Our findings pave the way for interventions that target the pathological stiffness of titin in disease.*

Pair 5: GPT

*This research provides crucial insights into how the structural arrangement of a viral genome within a virus influences its ability to infect host cells. The study reveals a temperature-dependent transition in the density of the DNA within the virus, which occurs near the typical body temperature of potential hosts. This transition facilitates the rapid ejection of the viral genome into a host cell, a critical step in viral infection. Understanding these mechanisms could potentially lead to the development of new strategies for preventing or treating viral infections.*

Pair 5: PNAS

*This work explains the structural origin of the temperature-dependent DNA density transition in bacteriophage λ capsid, occurring close to the physiological temperature favorable for infection (37 °C, human body temperature). Using small-angle neutron scattering, with contrast-matched scattering contribution from viral capsid proteins, we unveiled two coexisting DNA phases in a capsid—a hexagonally ordered high-density shell-DNA phase in the capsid periphery and a low-density, less-ordered DNA phase in the core. At the transition temperature, a density and volume transition occurs in the core-DNA, resulting in lower density and reduced packing defects. This yields increased mobility of the core-DNA phase, facilitating rapid DNA ejection events from phage into a host bacterial cell.*

# **Table S6: Descriptive Statistics and Correlation Matrix for Study 2**

| Variable | *M* | *SD* | 1 | 2 | 3 | 4 | 5 | 6 | 7 |
| --- | --- | --- | --- | --- | --- | --- | --- | --- | --- |
|  |  |  |  |  |  |  |  |  |  |
| 1. Intelligent | 5.07 | 1.05 | -- |  |  |  |  |  |  |
|  |  |  |  |  |  |  |  |  |  |
| 2. Credible | 4.60 | 1.16 | .65** | -- |  |  |  |  |  |
|  |  |  | [.61, .69] |  |  |  |  |  |  |
|  |  |  |  |  |  |  |  |  |  |
| 3. Trustworthy | 4.56 | 1.12 | .65** | .84** | -- |  |  |  |  |
|  |  |  | [.60, .68] | [.82, .86] |  |  |  |  |  |
|  |  |  |  |  |  |  |  |  |  |
| 4. AI | 3.89 | 1.44 | -.18** | -.20** | -.22** | -- |  |  |  |
|  |  |  | [-.24, -.11] | [-.27, -.14] | [-.29, -.16] |  |  |  |  |
|  |  |  |  |  |  |  |  |  |  |
| 5. Human | 4.55 | 1.39 | .24** | .28** | .33** | -.79** | -- |  |  |
|  |  |  | [.18, .30] | [.21, .34] | [.27, .39] | [-.82, -.77] |  |  |  |
|  |  |  |  |  |  |  |  |  |  |
| 6. Clear | 3.95 | 1.58 | .16** | .32** | .33** | -.26** | .31** | -- |  |
|  |  |  | [.10, .23] | [.25, .38] | [.27, .39] | [-.33, -.20] | [.25, .37] |  |  |
|  |  |  |  |  |  |  |  |  |  |
| 7. Complex | 4.74 | 1.41 | .21** | .03 | .01 | .14** | -.13** | -.55** | -- |
|  |  |  | [.14, .27] | [-.04, .10] | [-.06, .08] | [.07, .21] | [-.20, -.07] | [-.60, -.50] |  |
|  |  |  |  |  |  |  |  |  |  |
| 8. Understand | 3.46 | 1.55 | .05 | .23** | .25** | -.20** | .25** | .72** | -.56** |
|  |  |  | [-.02, .11] | [.17, .30] | [.19, .31] | [-.27, -.13] | [.18, .31] | [.69, .75] | [-.60, -.51] |
|  |  |  |  |  |  |  |  |  |  |

*Note*. ** *p* < .01. Numbers in brackets are 95% Confidence Intervals.

# **Verbatim Prompts for Study 3**

**Multiple Choice Solicitation**

*Read the following two summaries of an academic research article. Create one multiple choice question that could be answered by both summaries to test if a reader understood the basic premise of the science. Randomly choose the correct answer for the multiple choice question. Make four answer options for the question***.**

**Large Language Model Agreement on Multiple Choice Answers**

*Read the following two summaries of an academic research article. Then, based on these summaries, answer the multiple choice question. Please provide an answer.*

**Large Language Model Coding of Human Summaries**

*You will be presented with a text called Significance Statement. Read this statement, and then after, read a summary provided by someone who is trying to summarize this in their own words.*

*Grade this response on the following scale:*

- *0 points: no answer, an answer equivalent to I don't know, a simple restatement of the question, or an entirely or almost entirely incorrect answer,*
- *1 point: a partially accurate summary that includes some portions of the full summary but is either missing pieces or has additional incorrect information added,*
- *2 points: an answer that contains all or almost all of the elements of the full summary, with only minor omissions or inaccuracies.*

*Only provide a number — 0, 1, or 2 — based on your assessment and do not provide any other information.*

# **Table S7: Descriptive Statistics and Correlation Matrix for Study 3**

| Variable | *M* | *SD* | 1 | 2 | 3 | 4 | 5 | 6 | 7 | 8 | 9 | 10 |
| --- | --- | --- | --- | --- | --- | --- | --- | --- | --- | --- | --- | --- |
|  |  |  |  |  |  |  |  |  |  |  |  |  |
| 1. Intelligent | 5.15 | 1.23 |  |  |  |  |  |  |  |  |  |  |
|  |  |  |  |  |  |  |  |  |  |  |  |  |
| 2. Credible | 4.77 | 1.23 | .76** |  |  |  |  |  |  |  |  |  |
|  |  |  | [.73, .78] |  |  |  |  |  |  |  |  |  |
|  |  |  |  |  |  |  |  |  |  |  |  |  |
| 3. Trustworthy | 4.67 | 1.24 | .74** | .85** |  |  |  |  |  |  |  |  |
|  |  |  | [.71, .76] | [.84, .87] |  |  |  |  |  |  |  |  |
|  |  |  |  |  |  |  |  |  |  |  |  |  |
| 4. AI | 3.99 | 1.46 | -.11** | -.20** | -.21** |  |  |  |  |  |  |  |
|  |  |  | [-.16, -.06] | [-.25, -.14] | [-.27, -.16] |  |  |  |  |  |  |  |
|  |  |  |  |  |  |  |  |  |  |  |  |  |
| 5. Human | 4.43 | 1.41 | .24** | .33** | .34** | -.78** |  |  |  |  |  |  |
|  |  |  | [.19, .29] | [.28, .38] | [.29, .39] | [-.81, -.76] |  |  |  |  |  |  |
|  |  |  |  |  |  |  |  |  |  |  |  |  |
| 6. Clear | 3.65 | 1.66 | .23** | .34** | .36** | -.23** | .34** |  |  |  |  |  |
|  |  |  | [.18, .28] | [.29, .39] | [.31, .40] | [-.28, -.17] | [.29, .39] |  |  |  |  |  |
|  |  |  |  |  |  |  |  |  |  |  |  |  |
| 7. Complex | 4.97 | 1.51 | .22** | .06* | .06* | .12** | -.10** | -.50** |  |  |  |  |
|  |  |  | [.17, .27] | [.00, .11] | [.00, .12] | [.06, .17] | [-.16, -.05] | [-.54, -.46] |  |  |  |  |
|  |  |  |  |  |  |  |  |  |  |  |  |  |
| 8. Understand | 3.32 | 1.58 | .14** | .29** | .30** | -.18** | .28** | .77** | -.52** |  |  |  |
|  |  |  | [.09, .20] | [.23, .34] | [.24, .35] | [-.23, -.12] | [.23, .33] | [.75, .79] | [-.56, -.48] |  |  |  |
|  |  |  |  |  |  |  |  |  |  |  |  |  |
| 9. Comp. index | -0.00 | 1.52 | .03 | .08** | .06* | -.12** | .10** | .28** | -.24** | .32** |  |  |
|  |  |  | [-.02, .09] | [.02, .13] | [.01, .12] | [-.18, -.07] | [.04, .15] | [.23, .33] | [-.29, -.19] | [.27, .37] |  |  |
|  |  |  |  |  |  |  |  |  |  |  |  |  |
| 10. Mul. choice | 0.62 | 0.49 | -.04 | -.05 | -.06* | -.05 | .02 | .06* | -.08** | .07* | .76** |  |
|  |  |  | [-.10, .01] | [-.10, .01] | [-.11, -.00] | [-.10, .01] | [-.04, .07] | [.00, .11] | [-.13, -.02] | [.02, .13] | [.74, .78] |  |
|  |  |  |  |  |  |  |  |  |  |  |  |  |
| 11. Free resp. coding | 0.82 | 0.58 | .09** | .16** | .15** | -.14** | .13** | .37** | -.29** | .42** | .76** | .16** |
|  |  |  | [.04, .15] | [.11, .22] | [.10, .21] | [-.20, -.09] | [.07, .18] | [.32, .41] | [-.34, -.24] | [.38, .47] | [.74, .78] | [.10, .21] |
|  |  |  |  |  |  |  |  |  |  |  |  |  |

*Note*. Comp. index = comprehension index. Mul. choice = scores on the multiple choice question. Free resp. coding = average ratings from GPT-4o and GPT-4 on the free response coding.

# **References**

1. M. Foxman, D. M. Markowitz, D. Z. Davis, Defining empathy: Interconnected discourses of virtual reality’s prosocial impact: *New Media & Society* **23**, 2167–2188 (2021).

2. D. M. Markowitz, The meaning extraction method: An approach to evaluate content patterns from large-scale language data. *Frontiers in Communication* **6**, 13–13 (2021).

3. C. K. Chung, J. W. Pennebaker, Revealing dimensions of thinking in open-ended self-descriptions: An automated meaning extraction method for natural language. *Journal of Research in Personality* **42**, 96–132 (2008).
